# Anaphylaxis Following Human Prothrombin Complex Concentrate in a Child with Lupus Anticoagulant Hypoprothrombinemia Syndrome: A Cautionary Tale

**DOI:** 10.1055/s-0038-1624567

**Published:** 2018-01-29

**Authors:** Heshani Mediwake, Jeremy Robertson, Joanne Beggs, Jane Mason

**Affiliations:** 1 Department of Haematology, Pathology Queensland Central Laboratory, Royal Brisbane and Women's Hospital, Herston, Queensland, Australia; 2 School of Medicine, University of Queensland, St Lucia, Queensland, Australia; 3 Department of Haematology and Haemophilia, Lady Cilento Children's Hospital, South Brisbane, Queensland, Australia; 4 Queensland Haemophilia Centre, Royal Brisbane and Women's Hospital, Herston, Queensland, Australia

**Keywords:** coagulation factors, coagulation inhibitors, factor concentrates

## Abstract

A previously healthy 3-year-old girl presented with a short history of mucocutaneous bleeding and a spontaneous left knee hemarthrosis following a nonspecific viral gastroenteritis. Initial investigations for a bleeding disorder revealed a normal platelet count; however, coagulation studies revealed a prothrombin time (PT) of 25 seconds and an activated partial thromboplastin time (APTT) of 66 seconds (both prolonged). The APTT did not correct on mixing with normal plasma, and further testing confirmed the presence of a strong lupus anticoagulant (LA). One-stage assays of factor VIII, VII, and X were normal, but factor II was markedly reduced. Based on this distinct clinicopathological picture, a diagnosis of lupus anticoagulant hypoprothrombinemia syndrome (LAHS) was made. Due to the presence of a hemarthrosis, the patient was treated with clotting factor concentrate. Human prothrombin complex concentrate (PROTHROMBINEX-VF) was used as a source of factor II replacement; however, during the infusion the patient developed anaphylaxis necessitating resuscitation. The patient was observed without further factor replacement, and the bleeding symptoms resolved over several days. Within 3 weeks her PT and factor II had normalized but the APTT remained prolonged. After 6 months the coagulation profile had completely normalized and the LA was negative. It is unusual to require replacement of factor II in paediatric LAHS because bleeding is typically minor and self-limited. Anaphylaxis to clotting factor concentrates has not been previously reported in the context of LAHS, but is well described in patients with congenital factor IX deficiency (hemophilia B). Whilst the potential mechanism for anaphylaxis in our patient is unknown, it is recommended that human prothrombin complex concentrates should be used cautiously in paediatric LAHS.

The lupus anticoagulant hypoprothrombinemia syndrome (LAHS) is a very uncommon disorder and a rare exception to the widely understood premise that lupus anticoagulants (LAs) are not associated with bleeding. LAs in general comprise a heterogenous group of antibodies that are directed against phospholipids and thus affect the phospholipid-dependent activated partial thromboplastin time (APTT) test in vitro, but have no true in vivo anticoagulant effect. 1 Rarely, patients may present with laboratory evidence of a LA, along with an elevated prothrombin time (PT) secondary to antibody specificity against factor II, leading to a true deficiency and clinical bleeding symptoms. These constellation of findings constitutes the LAHS. 2

Anecdotally, we encounter approximately one to two cases of pediatric LAHS per year in our tertiary hospital. The classic presentation is of a young child with bruising following a recent virus. We typically take an observational approach in this setting, with follow-up to demonstrate normalization of the coagulation profile and factor II level along with disappearance of the LA.

We report here a highly unusual case of LAHS in a 3.5-year-old girl who presented with more significant bleeding necessitating replacement therapy and subsequently developed anaphylaxis to the human prothrombin complex concentrate PROTHROMBINEX-VF (CSL Behring, Broadmeadows, Victoria, Australia). Formal consent for publication of this case report was obtained.

The child was brought to the emergency department with a short history of bruising, left knee swelling, and a single episode of self-limiting epistaxis. One week prior to admission, she had confirmed adenovirus gastroenteritis. She had no significant past medical history and no history of atopy. Examination revealed widespread bruising, a small left knee hemarthrosis and an intensely pruritic urticarial type reaction to adhesive dressings.

Her full blood count revealed a mildly elevated platelet count of 582 × 109/L, with normal hemoglobin (116 g/dL) and white cell count (9.9 × 109/L) and an unremarkable film. A basic coagulation profile (ACL TOP Analyser) demonstrated a prolonged PT of 25 seconds (normal range: 9–13 seconds) and a prolonged APTT of 66 seconds (normal range: 24–39 seconds) which did not correct on a 50% mixing study with normal pooled plasma.

A factor VIII assay was performed using a single-stage APTT-based system ( Table 1 ) and demonstrated a normal result of 0.68 U/mL (0.5–1.5 U/mL). Factor II, VII, and X assays were performed using a single-stage PT-based system ( Table 1 ) and demonstrated a reduced factor II level of 0.09 U/mL, normal factor VII of 0.64 U/mL, and normal factor X of 0.90 U/mL (0.50–1.50 U/mL). LA testing in our laboratory is aligned with the ISTH SSC 2009 guidelines 3 and includes dilute Russell's viper venom time and APPT (screen and confirm) pathways. A strongly positive LA was detected.

**Table 1:** Methods used for coagulation assays

Assay	Method
APTT	ACL TOP Analyser, DG-FVIII deficient plasma; Diagnostic Grifols, Passeig Fluvial, Barcelona, Spain
PT	ACL TOP Analyser, HemosIL factor deficient plasmas; Instrumentation Laboratory, Bedford, Massachusetts, United States

The child was diagnosed with LAHS given the classic clinical/laboratory scenario. The factor II inhibitor level was not performed, as this was not clinically indicated and did not aid the diagnosis. A decision to use PROTHROMBINEX-VF was made given the high concentration of factor II units per milliliter, theoretically requiring less volume, perceived lower risk of adverse reactions, and negligible infectious risk (as compared with fresh frozen plasma [FFP]). Replacement of factor II was indicated as the child had a hemarthrosis. Following commencement of the infusion, she developed anaphylaxis necessitating adrenaline and fluid bolus support, and made a full recovery. A single doses of prednisone and promethazine were also subsequently administered. Given the noncritical nature of bleeding, a decision was made to manage expectantly, with no further plasma products transfused. Her bleeding symptoms and urticarial rash resolved over subsequent days and upon follow-up 3 weeks later, her PT (12 seconds) and factor II had normalized (0.97 U/mL), although the APTT remained prolonged (48 seconds) and the LA screen remained positive. At 6 months of follow-up, her coagulation profile was entirely normal and the LA screen was negative.

LAHS was initially described by Rapaport et al in 1960. 2 Bajaj et al later described the mechanism of hypoprothrombinemia in LAHS, by demonstrating the presence of antiprothrombin antibodies, which when bound to prothrombin are cleared rapidly from the circulation leading to hypoprothrombinemia. 4 Adult retrospective data report an apparent predominance in younger females and an association with autoimmune conditions, infections, and occasionally medications. 5 Bleeding is the most common presenting symptom, which is of variable severity ranging from minor epistaxis to intracerebral hemorrhage. 5 6 There are no standard recommendations on management and an individualized approach is clearly required depending on associated comorbid conditions and severity of bleeding. Postinfectious LAHS with minor bleeding is often self-limiting, 6 particularly in children, whereas noninfectious LAHS is more likely to relapse and require immunosuppressive therapy aimed at antibody eradication. The mainstay of immunosuppressive therapy is corticosteroids; however, use of other agents including azathioprine, cyclophosphamide, intravenous immunoglobulin, Rituximab, and plasma exchange are described. 5

Previous reports describing supportive therapy for treatment of bleeding have included the use of FFP, human prothrombin complex, vitamin K, and recombinant factor VIIa. Given the long half-life of prothrombin (median 60 hours) and nonneutralizing nature of the antibody, replacement of factor II (in the form of FFP or human prothrombin complex concentrate) is theoretically feasible (in contrast to most patients with acquired hemophilia A).

Our patient had a fairly classical initial presentation of LAHS with bleeding manifestations following adenovirus infection. It is unusual to have to treat postinfectious LAHS in children with factor replacement, as usually the only manifestation of bleeding is bruising, and it is a self-limited phenomenon. The development of an antibody with true specificity along with new atopic skin manifestations and subsequent anaphylaxis to PROTHROMBINEX-VF in this child are suggestive of more global immune dysregulation occurring post–viral infection, and to our knowledge this constellation of findings has not been reported previously. The higher concentration of factor II in the concentrate could be hypothesized to have some role in the pathophysiology of anaphylaxis in our case. Certainly, anaphylaxis to factor IX concentrate in children with congenital hemophilia B with inhibitors is well described, although the underlying immunological mechanisms remain unclear. 7

We could find only one case report of LAHS where human prothrombin complex concentrate was used with resolution of bleeding in an adult lupus nephritis patient. 8

We openly acknowledge that the mechanism for anaphylaxis in this scenario is not defined and it may be simply coincidental, but given the rarity of having to treat pediatric LAHS with factor replacement and the lack of pediatric reports of using human prothrombin complex in this group, this unusual side effect is relevant for future treatment decisions. Although it is unknown whether anaphylaxis would have occurred in our patient if administered FFP, given the overall rarity of both LAHS and reported anaphylaxis to PROTHROMBINEX-VF in other situations 9 we feel that human prothrombin complex concentrates should be used cautiously in LAHS, particularly in the pediatric group.
